# Developmental Toxicity of PEDOT:PSS in Zebrafish: Effects on Morphology, Cardiac Function, and Intestinal Health

**DOI:** 10.3390/toxics12020150

**Published:** 2024-02-15

**Authors:** Guan Yang, Dongzhi Gou, Ling-Kang Bu, Xing-Yi Wei, Huan Hu, Wen-Bo Huo, Marriya Sultan, De-Sheng Pei

**Affiliations:** 1 College of Architecture and Urban Planning, Chongqing Jiaotong University, Chongqing 400074, China; 2 Chongqing Institute of Green and Intelligent Technology, Chongqing School of University of Chinese Academy of Sciences, Chinese Academy of Sciences, Chongqing 400714, Chinahuowenbo@cigit.ac.cn (W.-B.H.);; 3 School of Public Health, Chongqing Medical University, Chongqing 400016, China

**Keywords:** developmental toxicity, histopathological image analysis, oxidative stress, transcriptomic analysis, zebrafish

## Abstract

Poly(3,4-ethylenedioxythiophene):poly(styrenesulfonate) (PEDOT:PSS) is a conductive polymer commonly used in various technological applications. However, its impact on aquatic ecosystems remains largely unexplored. In this study, we investigated the toxicity effects of PEDOT:PSS on zebrafish. We first determined the lethal concentration (LC_50_) of PEDOT:PSS in zebrafish and then exposed AB-type zebrafish embryos to different concentrations of PEDOT:PSS for 120 h. Our investigation elucidated the toxicity effects of zebrafish development, including morphological assessments, heart rate measurements, behavioral analysis, transcriptome profiling, and histopathological analysis. We discovered that PEDOT:PSS exhibited detrimental effects on the early developmental stages of zebrafish, exacerbating the oxidative stress level, suppressing zebrafish activity, impairing cardiac development, and causing intestinal cell damage. This study adds a new dimension to the developmental toxicity of PEDOT:PSS in zebrafish. Our findings contribute to our understanding of the ecological repercussions of PEDOT:PSS and highlight the importance of responsible development and application of novel materials in our rapidly evolving technological landscape.

## 1. Introduction

Energy is the foundation of human survival and the fundamental driving force behind economic growth and societal development. Currently, around 90% of the world’s energy comes from non-renewable fossil fuels. Global energy consumption is expected to increase at an average annual rate of 48% from 2012 to 2040 [1]. This underscores the urgent need for more efficient energy utilization strategies and technologies. Poly(3,4-ethylenedioxythiophene):poly(styrenesulfonate) (PEDOT:PSS) is a promising material that can help address our energy challenges. However, we must investigate its safety thoroughly to ensure sustainable use. In our rapidly advancing technological landscape, responsible development and implementation are crucial. The conducting polymer solution PEDOT, which was synthesized by the German company Bayer in 1988, is a combination of PEDOT and PSS. PEDOT itself is insoluble, which greatly limits its practical applications. In 1991, Bayer successfully created a stable and soluble form of PEDOT by combining it with PSS [2], which is now known as PEDOT:PSS. PEDOT:PSS is a material with remarkable properties, including the ability to form strong films, high optical transparency, excellent electrical conductivity, and environmental stability [2]. This material has been used in various fields [3,4,5], such as solar cells, light-emitting diodes, supercapacitors, and antistatic coatings. Moreover, it has great potential for applications in wearable smart devices, biomedical sensors, tissue engineering, neural interfaces, and other areas [6,7,8].

The use of PEDOT:PSS has become widespread due to its water solubility [9]; however, there is a significant possibility of it leaking into the environment, raising concerns regarding its safety. The properties of PEDOT:PSS, such as its high environmental stability and its ability to interact with ions in the surrounding environment, suggest that it could potentially have an impact on aquatic ecosystems. A recent study has reported that PEDOT:PSS is cytotoxic and can affect cellular transcription [10]. Another study indicates that it also negatively impacts algae in water bodies, inhibiting algal growth and reducing chlorophyll production [11]. Despite its potential, there is a notable gap in the research concerning the toxicological impact of PEDOT:PSS on aquatic species. To address this, our study delves into the developmental toxicity of PEDOT:PSS, utilizing zebrafish as a representative model organism.

The zebrafish (*Danio rerio*) is a tropical freshwater fish that belongs to the minnow family, *Cyprinidae*, in the order *Cypriniformes*. It is native to countries like India and Bangladesh. Adult zebrafish have a body length of 3–4 cm and reach sexual maturity at around 3 months. They require low water quality and thrive at an optimal temperature of 28.5 °C. Males have a slender body shape, while females have a larger abdomen and an overall bulkier physique, exhibiting distinct sexual dimorphism [12]. The zebrafish is a commonly used model organism in genetics, developmental biology, and toxicology due to its various advantageous characteristics. These include its small size, ability to produce a large number of eggs, external fertilization, fast development, transparent embryos for easy observation, and easy maintenance in laboratory settings [13,14]. It is worth mentioning that zebrafish and humans share many similarities since both are vertebrates [15]. The sequencing of the zebrafish genome was completed in 2009, and it was found that it has 87% similarity to the human genome. The gene and protein sequences and functions are highly conserved between the two species, which indicates that the results of drug toxicity tests conducted on zebrafish can often be applied to humans as well [16,17].

In this study, zebrafish embryos were exposed to different concentrations of PEDOT:PSS solution and related experiments were conducted at different stages of development to study its toxicity effect. This study represents the first comprehensive exploration of PEDOT:PSS developmental toxicity in zebrafish and enhances our understanding of the ecological consequences associated with PEDOT:PSS.

## 2. Materials and Methods

### 2.1. Ethics Statement

All experiments involving zebrafish (*Danio rerio*) were conducted in accordance with the “Guide for the Care and Use of Laboratory Animals” (Eighth Edition, 2011. ILARCLS, National Research Council, Washington, DC, USA) and the Laboratory Animal Welfare standard of the Chinese National Standard GB/T 35892-2018 [18].

### 2.2. Husbandry and Zebrafish Egg Production

The AB strain of wild-type zebrafish was obtained from the Institute of Hydrobiology, Chinese Academy of Sciences. They were bred and maintained in our laboratory and fed three times daily. The light-dark cycle was set to 14 h of light and 10 h of darkness, while the temperature was consistently maintained at 28.0 ± 1 °C. Before commencing the experiment, adult zebrafish were placed in a breeding tank with a sex ratio of 1 female to 2 males, separated by dividers. The following morning, the embryos were collected within 1 h of spawning and preserved in fresh egg water containing 2 mg/L of methylene blue. At 4 h post-fertilization (hpf), the embryos were observed under a dissecting microscope, and only those displaying normal development were selected for the subsequent experiments.

### 2.3. Chemicals

PEDOT:PSS (2.8% *w*/*w*) was purchased from a Sigma Aldrich (Shanghai, China) distributor in China (Cat. 560596, CAS 155090-83-8). Analytical-grade reagents were used throughout the study.

### 2.4. Measurement of Zebrafish Developmental Toxicity and LC_50_ of PEDOT:PSS

**Figure 1:**
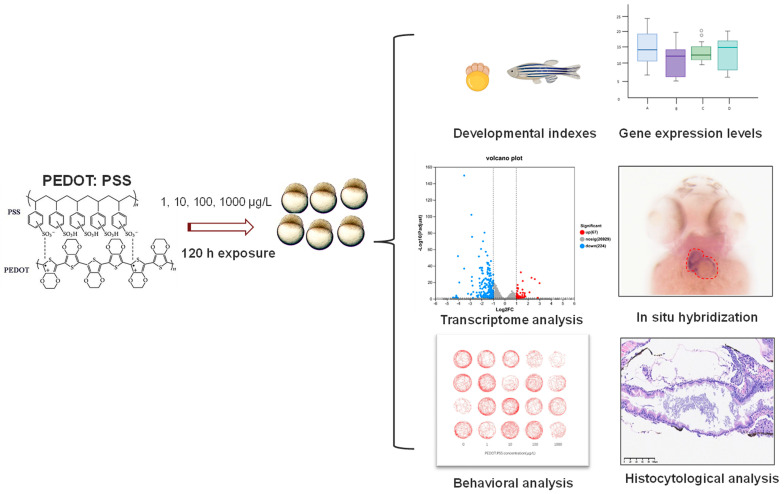
The experimental design for investigating toxicity effects of PEDOT:PSS in zebrafish.

The zebrafish development parameters, including body length, body weight, and heart rate, were determined according to previous reports [19,20]. Figure 1 shows the experimental design for investigating the toxicity effects of PEDOT:PSS in zebrafish in this study. Our experiment consisted of a blank control group and experimental groups with PEDOT:PSS concentrations of 1, 10, 100 μg/L, and 1 mg/L according to our pre-experiment results of zebrafish’s viability data. In this experiment, we exposed 50 zebrafish embryos at 4 hpf to different concentrations of PEDOT:PSS for 120 h in 10 cm diameter Petri dishes (*n* = 50), with 20 mL of solution in each group. We set up three replicate groups for each concentration and replaced half of the exposure solution in each group every 24 h. Any dead or abnormal embryos/larvae were promptly recorded and removed. Based on the recorded results, we calculated the LC_50_ of PEDOT:PSS.

### 2.5. Measurement of Heart Rate and Other Basic Parameters

At the 48 hpf stage, we selected 10 zebrafish per group (n = 10), all morphologically healthy under a stereomicroscope, for heart rate measurement. The zebrafish were acclimatized for over 15 min in an indoor setting before observation. Under the stereomicroscope, we focused on the zebrafish’s heart area until the heartbeat was clearly visible. We counted the beats for one minute using a handheld counter. The recorded data were then analyzed using Graph Prism 9.0 software. The measurements for weight and body length are taken at 120 hpf, the heart rate is determined at 48 hpf, and the hatching rate is measured at 72 hpf (*n* = 50).

### 2.6. Behavioral Experiments

We utilized the Danio Vision™ system (Noldus, Wageningen, The Netherlands) to evaluate the swimming behavior of zebrafish larvae. After a 120-h exposure period, unaffected larvae were randomly selected from each group and placed into individual wells of a 24-well plate, with one larva per well. The experimental water temperature was maintained at 28 ± 0.5 °C. The larvae were then placed into a tracking chamber, and their swimming behavior was recorded for 10 min at a frequency of 25 frames per second. The photo motor response was also recorded. In the inverted photo motor response trials, baseline activity was assessed for a duration of 10 s, succeeded by a 1 s exposure to darkness (less than 15 lux), then followed by exposure to bright white light once again. The experiment was repeated five times. Finally, the EthoVision XT^®^ computer tracking software (version 17.5) from Noldus was employed to analyze and plot the movement trajectories of each larva, as well as to collect data on total distance traveled, speed, acceleration, mobility state, photo motor response, and activity levels. Video analysis and data collection were performed using this software.

### 2.7. Expression Level Analysis of Oxidative Stress-Related Genes

Oxidative stress is a biochemical reaction and leads to a series of biological effects and cell damage [21,22,23]. We selected ten genes related to oxidative stress and the *gapdh* gene as the internal reference gene. We extracted the total RNA of 30 zebrafish larvae from each group using RNAisoPlus reagent (Takara Bio Inc., Shiga, Japan) according to the manufacturer’s instructions. The quality of total RNA was measured using a spectrophotometer and 1% agarose gel electrophoresis. Next, we synthesized cDNA using the Primer Script RT kit (Takara Bio, Shiga, Japan) with 1 μg RNA of each sample following the manufacturer’s instructions. qRT-PCR was performed using the ABI 7300 system (PerkinElmer Applied Biosystems, Shelton, CT, USA) with the TB Green PCR kit (Toyobo, Tokyo, Japan). The primers of zebrafish were designed using the NCBI Primer online tool (The primer sequences can be seen in the SI). The PCR program was as follows: 95 °C for 30 s, 95 °C for 20 s, 58 °C for 20 s, and 72 °C for 45 s for 40 cycles. At the end of the amplification process, we achieved the dissociation curve with 95 °C for 15 s, 60 °C for 1 min, and 95 °C for 15 s. We performed three replicates of all groups and used the 2^−ΔΔCT^ method to quantify the target gene expression levels.

### 2.8. In Situ Hybridization Experiment

For the in situ hybridization experiments, zebrafish larvae at 72 hpf were selected from the control group, and the group was exposed to PEDOT:PSS at a concentration of 1 mg/L (*n* = 20). The vector pEGM-TEasy, which contains T7 and SP6 promoters, was used. To prepare the cardiac myosin light chain 2 (*cmlc2*) and natriuretic peptide a (*nppa*) probes, primer sequences were used (Table S1). Antisense RNA probes were used for in situ hybridization following our procedures described previously [24]. The imaging process utilized the Nikon NIS-Elements platform (Nikon Metrology Inc., Boston, MI, USA) for capturing images, while the Bandscan V 5.0 software (Bio-Rad, Hercules, CA, USA) was employed to analyze signal intensity.

### 2.9. Histocytological Analysis of Zebrafish Larvae

To study the effects of PSS exposure, 5 zebrafish larvae at 120 hpf were chosen from the exposure group and 5 from the blank control group. Both groups were euthanized with 80 mg/L MS-222 and fixed in 4% formaldehyde for 24 h. After dehydration in ethanol, the larvae were embedded in paraffin and sliced with a 5 μm-thick slicing machine. The larval sections of both groups were stained with Hematoxylin and Eosin (H&E) and examined for morphology using a Nikon microscope and NIS software (NIS-Elements BR 4.30.00).

### 2.10. Transcriptomic Analysis

After 120 h exposure, we chose 30 zebrafish from each of the blank control groups and the exposure groups (*n* = 30) with a concentration of 1 mg/L PEDOT:PSS because of obvious defect phenotypes. Three parallel experiments were conducted at each concentration, resulting in a total of 90 zebrafish for each concentration. The zebrafish were quickly frozen in liquid nitrogen and stored at −80 °C. RNA sequencing was completed by Meiji Company in Shanghai, China. Genes with an adjusted *p*-value (padj) of less than 0.05 and a fold change (FC) of more than 2 were defined as differentially expressed genes (DEGs). The R3.2.2 script was used to conduct the Kyoto Encyclopedia of Genes and Genomes (KEGG) analysis, while Goatools was used to conduct the Gene Ontology (GO) enrichment analysis. Pathways with an adjusted *p*-value (padj) of less than 0.05 were considered significant pathways.

### 2.11. Statistical Analysis

We used a one-way analysis of variance (ANOVA) to calculate the differences between the variables. The diagrams were analyzed using GraphPad 8.0 software (GraphPad Software, San Diego, CA, USA). The data were expressed as means ± standard error (SEM). Differences between groups and treatments were considered significant when *p* < 0.05.

## 3. Results

### 3.1. Developmental Toxicity Effects after Exposure to PEDOT:PSS

**Figure 2:**
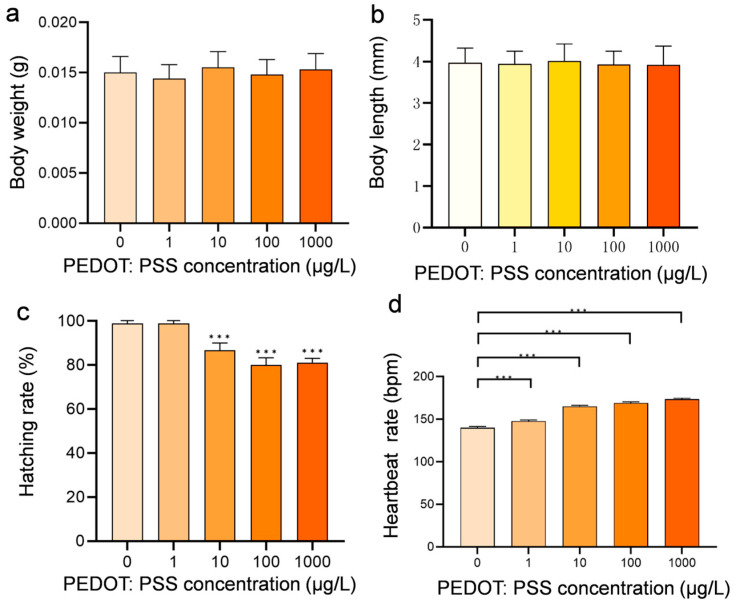
The developmental indexes of zebrafish after exposure to different concentrations of PEDOT:PSS. The body weight (**a**) and body length (**b**) of zebrafish larvae were measured at 120 hpf. The hatching rate (**c**) and heartbeat rate (**d**) of zebrafish larvae were recorded at 72 and 48 hpf, respectively. Each experiment was repeated three times, and the results are presented as mean ± standard deviation. Statistical analysis showed that there was a significant difference in heartbeat rate between the control group and the exposed group (*** *p* < 0.001).

The body weight and body length of zebrafish larvae were analyzed after 120 h exposure. Interestingly, there was no obvious change in body weight and body length (Figure 2a,b). Then, we measured the LC_50_ of PEDOT:PSS after exposure to different concentrations of solutions and found that the LC_50_ value was 24.52 mg/L. In this study, zebrafish embryos were exposed to concentrations below 1 mg/L, and no significant mortality cases were observed, even at the highest concentration used (1 mg/L). However, a few zebrafish in the higher concentration group showed deformities, but the ratio is very low (less than 10%). Although PEDOT:PSS exhibited an inhibitory effect on the hatching of zebrafish embryos (Figure 2c), it did not affect the overall hatching success. The heart rate tests conducted after 48 h exposure showed that the heartbeat rate of zebrafish increased with the concentration of PEDOT:PSS (Figure 2d), indicating that PEDOT:PSS has a certain effect on the cardiac function of zebrafish.

### 3.2. Effects of PEDOT:PSS on Oxidative Stress-Related Gene Expression

**Figure 3:**
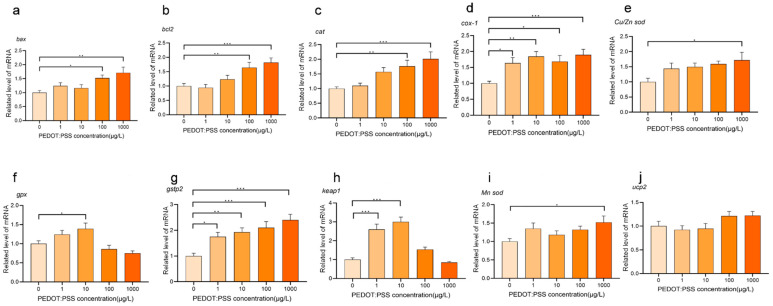
Expression levels of genes related to oxidative stress in zebrafish. (**a**–**j**) are the expression levels of *bax*, *bcl2*, *cat*, *cox-1*, *Cu/Zn sod*, *gpx*, *gstp2*, *keap1*, *Mn sod*, and *ucp2* after exposure to different concentrations of PEDOT:PSS, respectively. The experiment was repeated three times, and the results were expressed as mean ± standard deviation. The findings indicate a statistically significant difference between the control and exposure groups, with * *p* < 0.05, ** *p* < 0.01, and *** *p* < 0.001.

The expression of genes related to oxidative stress differed at all experimental concentrations when compared to the control group, indicating that PEDOT:PSS induces a certain level of oxidative stress in zebrafish larvae (Figure 3). As the concentration of PEDOT:PSS increased, most of these genes showed a greater difference in their relative expression levels from the control group (Figure 3). This suggests that the impact of PEDOT:PSS on oxidative stress within the zebrafish larvae becomes more pronounced with higher concentrations, implying a positive correlation between overall oxidative stress levels in zebrafish larvae and PEDOT:PSS concentration.

### 3.3. Transcriptomic Analysis of Zebrafish Larvae after Exposure to PEDOT:PSS

**Figure 4:**
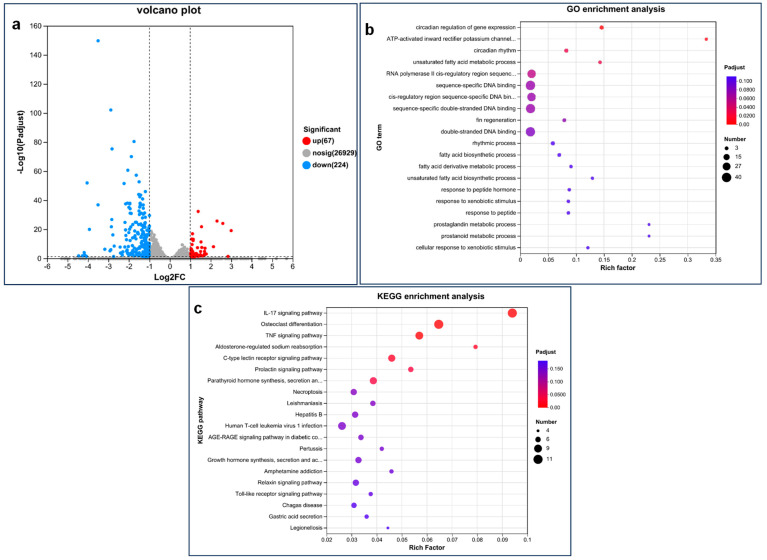
Transcriptome analysis of zebrafish at 120 hpf after exposure to 1 mg/L PEDOT:PSS. (**a**) The results were presented in a volcano diagram, where the red and blue dots represent significantly up- and downregulated genes, respectively. The grey dots signify no significant gene expression changes. (**b**,**c**) Our study identified the top 20 GO terms (**b**) and KEGG pathways for enrichment level (**c**).

Six sets of samples were analyzed, which altogether produced 46.32 GB of clean data. Each sample had at least 6.38 GB of clean data, and Q30 bases accounted for over 93.31%. The alignment efficiency of the clean readings of each sample using the specified reference genome ranged from 92.27% to 92.98%. These results show that the data quality is suitable for further analysis. Analysis revealed 291 significantly differentially expressed genes between the control group and the 1 mg/L exposure group, out of which 67 were upregulated and 224 were downregulated (Figure 4a). Then, GO enrichment analysis and further KEGG enrichment analysis were performed on all DEGs with significant changes (Figure 4b,c). The DEG is significantly enriched in several GO terms, including circadian regulation of gene expression, inward rectifier potassium channel activity activated by ATP, circadian rhythm, and unsaturated fatty acid metabolism processes (Table S2). Additionally, the significant enrichment of DEG involves several KEGG pathways: IL-17 signaling pathway, osteoclast differentiation, TNF signaling pathway, aldosterone-regulated sodium reabsorption, C-type lectin receptor signaling pathway, prolactin signaling pathway, and the synthesis, secretion, and action of parathyroid hormone (Table S3).

### 3.4. Concentration-Dependent Effects of PEDOT:PSS on Zebrafish Behavior

**Figure 5:**
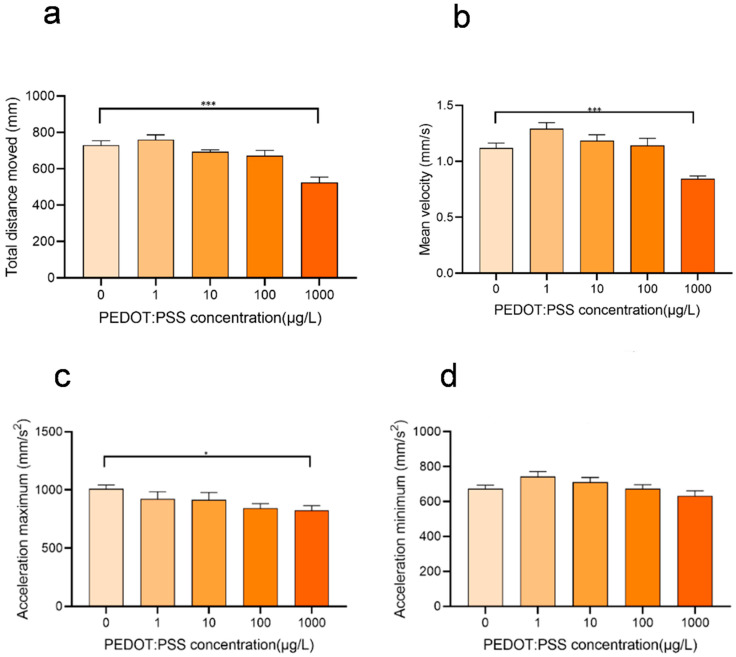
The results of the behavioral tests conducted on zebrafish at 120 hpf. (**a**) Total distance movement; (**b**) Mean velocity; (**c**) Maximum acceleration; (**d**) Minimum acceleration. The experiment was repeated five times, and the results are expressed as mean ± standard deviation. There was a statistically significant difference between the control and exposure group with * *p* < 0.05 and *** *p* < 0.001.

When exposed to low concentrations of PEDOT:PSS, zebrafish showed increased movement distance, velocity, acceleration, and activity compared to the control group that was not exposed. Behavioral trajectory and behavior hot zone analysis also indicated that their activity level was higher (Figure 5). However, this increase in activity is attributed to biological behavioral stress, where the organisms respond to environmental stimuli by evasive behaviors and attempt to find suitable areas for survival, and the intrinsic physiological processes and states of organisms are not greatly affected. Moreover, high concentrations of PEDOT:PSS (100, 1000 μg/L) cause zebrafish to prefer to stay still. When exposed to high concentrations of PEDOT:PSS, zebrafish showed decreased movement distance, velocity, acceleration, and activity compared to the control group. Behavioral trajectory and behavior hot zone analysis also showed reduced activity levels, and there was a trend of increasing inhibition of zebrafish activity with higher concentrations of PEDOT:PSS (Figure 5).

### 3.5. PEDOT:PSS Exposure Impairs Cardiac Development

**Figure 6:**
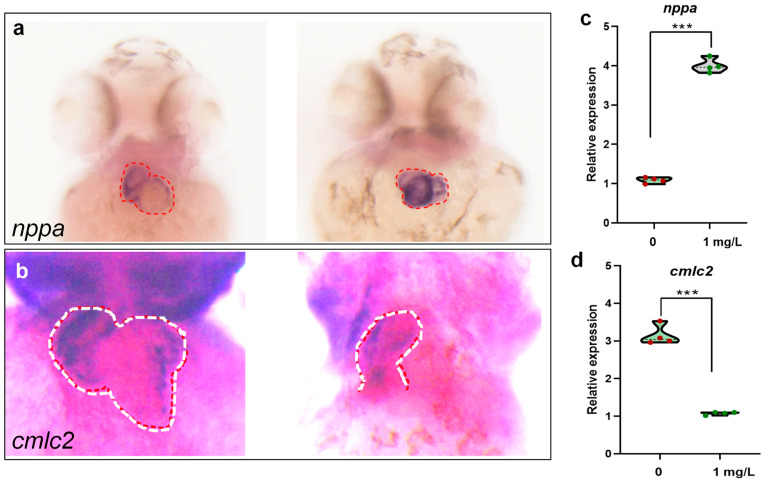
In situ hybridization results of *nppa* (**a**) and *cmlc2* (**b**). Control on the left, 1 mg/L PEDOT:PSS treatment on the right for both *nppa* and *cmlc2*. The region of interest is denoted by red or white circles. (**c**,**d**) The semi-quantitative results of (**a**,**b**). The red and white dash lines indicated the signal regions. There was a statistically significant difference between the control and exposure group with *** *p* < 0.001.

The results of the study showed that compared to the control group, the exposed group of zebrafish had significantly increased expression levels of *nppa* in their hearts (Figure 6a,c), suggesting that exposure to PEDOT:PSS can lead to heart failure in zebrafish larvae to some extent. Additionally, the expression of *cmlc2* in the hearts of the exposed zebrafish group indicates abnormalities (Figure 6b,d). This means that zebrafish larvae exposed to PEDOT:PSS have defects in the heart looping process, resulting in incomplete cardiac looping and affected cardiac development.

### 3.6. Detrimental Effects of PEDOT:PSS on Intestinal Cells

**Figure 7:**
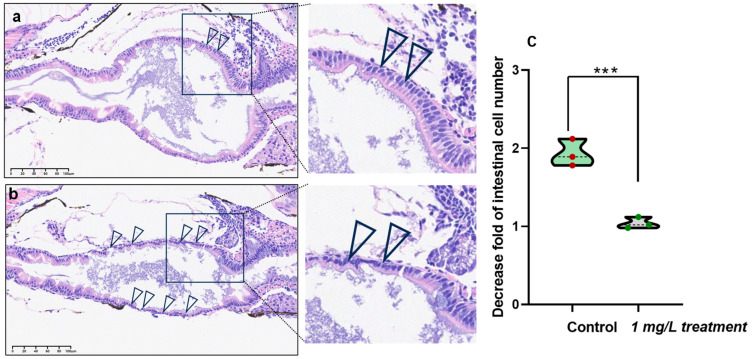
Visualization of intestinal cell defects in zebrafish after exposure to 1 mg/L PEDOT:PSS. At 120 hpf, the entire zebrafish intestinal tract was analyzed and visualized for control groups (**a**) and target groups treated with 1 mg/L PEDOT:PSS (**b**). The defects of intestinal cells are indicated by arrows. (**c**) The quantitative result of the decreased value of intestinal cells. There was a statistically significant difference between the control and exposure group with *** *p* < 0.001.

Our results demonstrate that exposure to PEDOT:PSS has harmful effects on intestinal cells. These effects include cellular shrinkage, a reduction in structural integrity, and a decrease in cellular population (Figure 7a,b). The intestinal cells of zebrafish larvae exposed to PEDOT:PSS were visibly smaller than those in the control group, indicating that the cells had undergone stress or damage due to the presence of PEDOT:PSS. Furthermore, the thinning of these cells indicates a possible alteration in cellular architecture, which could have a significant impact on cellular functionality and the overall robustness of the intestinal cells. We also found a significant decrease in the number of intestinal cells after exposure to PEDOT:PSS, suggesting that the PEDOT:PSS has adverse effects on cellular viability and proliferation within the intestinal environment of the zebrafish larvae, possibly leading to cellular death or impeded cellular division. These findings provide compelling evidence that exposure to PEDOT:PSS has a discernible impact on the intestinal cells of zebrafish larvae, with a trifecta of shrinkage, thinning, and reduced cellular numbers serving as a testament to the potential cellular responses, including stress, toxicity, or perturbations in cellular functionality.

## 4. Discussion

**Figure 8:**
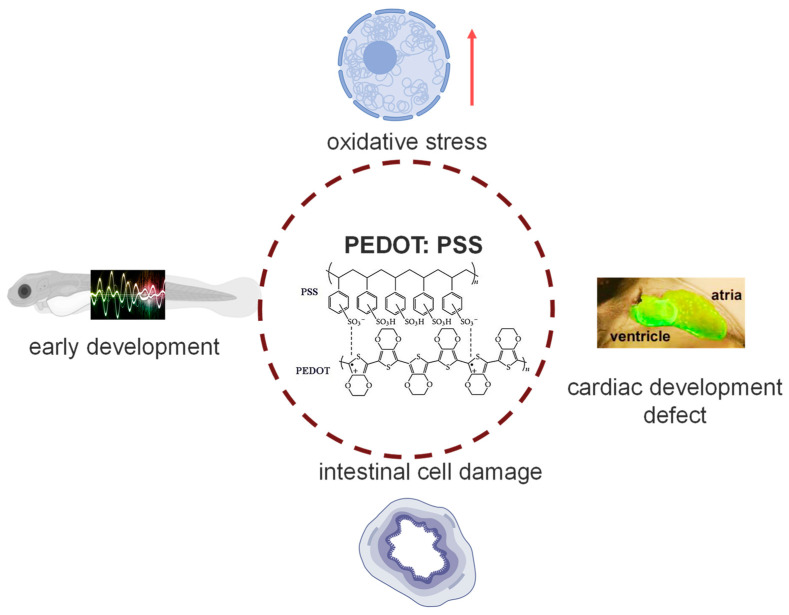
Summary of toxicity effects of PEDOT:PSS in zebrafish.

The widespread use of PEDOT:PSS in different fields has raised concerns about its safety. Previous studies have shown that it can have toxic effects on cells and algae in the water, affecting their physiological functions [10,11]. As it can easily enter the water body, we chose to study its toxicity in zebrafish, which is a more advanced organism compared to cells and algae, with a complete physiological system. The results obtained from this study are more practical and valuable (Figure 8).

We have calculated the LC_50_ data of PEDOT:PSS on zebrafish after exposure PEDOT:PSS for 96 h (Figure S1), which showed a concentration of 24.52 mg/L. To obtain more practical and valuable results, we set the experimental concentration as low as possible in an unknown environmental concentration. We referred to the literature [19] and began our experiment at a concentration of 1 µg/L, with a concentration gradient of 10 times. The highest experimental concentration in our study was 1 mg/L. In our experiment, we found that all concentrations of PEDOT:PSS solution exposed to zebrafish at 48 hpf had a higher heart rate compared to the control group. Additionally, we observed a positive correlation between the heart rate and the increase in concentration. This increase in heart rate is like the heart rate of zebrafish exposed to similar external substances [25,26].

The antiapoptotic B-cell lymphoma 2 (*bcl2*) and proapoptotic protein Bcl-2 Antagonist X (*Bax*) of the intrinsic pathway are positioned upstream in the sequence, leading to the mitochondrial generation of oxidative stress-triggering reactive entities [27]. Other genes including catalase (*cat*), cyclooxygenase (*cox*), Cu/Zn or Mn superoxide dismutases (*sod*), glutathione peroxidases (*gpx*), glutathione S-transferase Pi 2 (*gstp2*), kelch-1ike ECH-associated protein l (*keap1*), uncoupling protein 2 (*ucp2*), are all closed related to oxidative stress. Thus, they are selected to detect the level of oxidative stress, and the results indicate a direct relationship between the intensity of oxidative stress in zebrafish larvae and the concentration of PEDOT:PSS.

After analyzing the results from the zebrafish behavior experiments, we observed that low concentrations of PEDOT:PSS cause zebrafish larvae to move more compared to the control group. However, at high concentrations, it inhibits their movement. Our hypothesis is that low concentrations of PEDOT:PSS may make zebrafish more sensitive to stimulating substances in the environment, which triggers a stress response, and thus leads to increased movement data. Conversely, high concentrations of PEDOT:PSS may cause internal damage to zebrafish, ultimately leading to decreased mobility [28].

Nppa is a gene that marks myocardial hypertrophy and heart failure [29,30]. The results indicate that the expression of the *nppa* gene is increased in the group treated with PEDOT:PSS. It is worth noting that an increase in heart rate is one of the manifestations of heart failure [31], which is consistent with the increase in heart rate observed in zebrafish treated with PEDOT:PSS. This suggests that PEDOT:PSS may cause early heart failure in zebrafish to some degree. From the results, it can be seen that there are abnormalities in the heart looping process and impaired heart development in zebrafish treated with PEDOT:PSS. It is worth noting that exposure of zebrafish to exogenous substances that cause damage to the heart and intestinal tract is common in previous studies [32,33]. The *cmlc2* gene plays a crucial role in the development and function of the heart muscle [34], particularly in the formation of the sarcomere structure, which is the basic unit of muscle contraction. Loss of *cmlc2* leads to a disruption in sarcomere structure and compromises cardiac function [35]. In this study, exposure to PEDOT:PSS decreases the expression level of *cmlc2*, implying cardiac damage.

The use of PEDOT:PSS was found to cause damage to the intestines of zebrafish, as observed through HE staining of slices. Although there is limited specific research on the impact of PEDOT:PSS on the intestinal cells of zebrafish larvae, our study demonstrates that this damage leads to atrophy and thinning of the intestinal cavity, as well as a scattered arrangement of the intestinal wall cells. It has been previously observed in many studies that exposing zebrafish to exogenous substances often results in damage to their intestines [36,37,38].

Our analysis of the transcriptome revealed that 291 genes showed significant differential expression between the group that was exposed to 1 mg/L treated group and the control group. Out of these genes, 67 were upregulated, while 224 were downregulated. We then performed GO enrichment analysis and KEGG pathway analysis on all the DEGs that were significantly changed. Our analysis revealed that several GO terms were significantly enriched by the DEGs, including the regulation of gene expression circadian rhythm, ATP-activated inward rectifier potassium channel activity, circadian rhythm, and unsaturated fatty acid metabolic process. Moreover, several KEGG pathways were significantly enriched by the DEGs, including the IL-17 signaling pathway, osteoclast differentiation, TNF signaling pathway, aldosterone-regulated sodium reabsorption, C-type lectin receptor signaling pathway, prolactin signaling pathway, and parathyroid hormone synthesis and secretion.

The interleukin-17 (IL-17) family is a group of cytokines produced by different immune cells. It has multiple members, including IL-17A, IL-17B, IL-17C, IL-17D, IL-17E, and IL-17F. The IL-17 pathway is vital to the immune system’s function and plays a crucial role in the immune response. IL-17 can stimulate the production of pro-inflammatory cytokines, such as tumor necrosis factor-α (TNF-α), interleukin-1β (IL-1β), and interleukin-6 (IL-6), which can trigger inflammatory responses, attract immune cells into damaged areas, and respond to infections, injuries, or other inflammatory stimuli. It can also enhance the immune response to bacteria, fungi, and other microorganisms by stimulating epithelial cells to produce antimicrobial proteins, increasing mucosal immunity, and enhancing the activation and proliferation of T cells. However, an overactive IL-17 pathway can lead to autoimmune diseases, such as psoriasis and rheumatoid arthritis. These diseases may be related to abnormal immune responses caused by IL-17-mediated inflammatory reactions. Overall, the IL-17 pathway plays a crucial role in immune response, inflammatory response, anti-infection, and immune balance [39,40].

KEGG pathways in this study were also significantly enriched tumor necrosis factor (TNF), which is a cytokine produced by immune cells, such as macrophages, lymphocytes, and dendritic cells. TNF participates in physiological and pathological processes such as inflammatory response, cell survival, and cell apoptosis. TNF has two forms, TNF-α and TNF-β, of which TNF-α is the most studied type [41]. The TNF signaling pathway plays a crucial role in osteoclast differentiation and bone metabolism regulation. TNF-α promotes the differentiation of osteoclast precursor cells into mature osteoclasts by activating the RANKL (receptor activator of nuclear factor κB ligand) and its receptor RANK signal pathway. It also controls the survival and apoptosis of osteoclasts, maintaining normal levels of osteoclasts in vivo. This plays an important role in bone remodeling and maintaining bone tissue homeostasis. However, an overactive TNF-α signaling pathway can cause excessive differentiation and activation of osteoclasts, leading to disorders in bone metabolism, decreased bone density, and the occurrence of bone-related diseases [42]. Overall, the TNF signaling pathway can regulate osteoclast differentiation and affect individual bone health and balance.

Overall, the genes that were expressed differently between the treatment and control groups are significantly enriched in pathways that are related to immune regulation, inflammation response, bone development, blood pressure, and body fluid balance. These pathways are interlinked and have an impact on each other. This suggests that the zebrafish in the treatment group show certain abnormalities in these aspects. As a result, there is a high possibility of these fish developing related diseases in the future.

## 5. Conclusions

Our study indicates that exposure to PEDOT:PSS hurts the early development of zebrafish. qPCR results showed that genes related to oxidative stress changed after exposure. Behavioral studies indicate that low concentrations of PEDOT:PSS stimulate zebrafish movement, while high concentrations inhibit it. Heart rate testing and in situ hybridization confirm that PEDOT:PSS can cause damage to the heart development of zebrafish. Zebrafish larvae body section also indicates that PEDOT:PSS can cause certain damage to the gut of zebrafish. Transcriptome sequencing results show that after PEDOT:PSS treatment, zebrafish are at risk of immune regulation, bone health, and blood pressure balance regulation. The results of this study (Figure 8) supplement the insufficient physical information on PEDOT:PSS in aquatic poisoning and provide a theoretical basis for the environmental quality standards, drug regulation, and risk assessment of PEDOT:PSS.

## Data Availability

Data will be made available on request.

## Supplementary Materials

The following supporting information can be downloaded at https://www.mdpi.com/article/10.3390/toxics12020150/s1, Table S1: The primer sequences used in this study, Table S2: Significantly enriched GO terms after exposure to PEDOT: PSS, Table S3: Significantly enriched GO terms after exposure to PEDOT: PSS, Figure S1. The curve showing the LC50 value of zebrafish larvae after exposure to PEDOT: PSS for 96 h.
