# Supplementary material for: Developmental Toxicity of PEDOT:PSS in Zebrafish: Effects on Morphology, Cardiac Function, and Intestinal Health
Source: Toxics. 2024 Feb 15;12(2):150. doi: 10.3390/toxics12020150 (PMC10892323; doi:10.3390/toxics12020150)
Supplement: Supplementary file 1 [file toxics-12-00150-s001.zip › toxics-2820551-supplementary.pdf]

Table S1. The primer sequences used in this study

Genes	Primer sequences（5'→3'）
bax	F: GGCTATTTCAACCAGGGTTCC R: TGCGAATCACCAATGCTGT
gpx	F: AGATGTCATTCCTGCACACG R: AAGGAGAAGCTTCCTCAGCC
bcl2	F: AACCCAAATTCTGCGCAACG R: ATCTACCTGGGACGCCATCT
ucp2	F: CAAGGGGTTCATGCCATCCT R: GCTCAACTGGAACTGCATGC
cox-1	F: GACTACCCAGACGCCTATGC R: GAGGGCAGCCGTGTAATCAT
gstp2	F: TCTGGACTCTTTCCCGTCTCTC R: ATTCACTGTTTGCCGTTGCCGT
keap1	F: TGGATAACTACCTCTATGCCGT R: CCTTGGTTAAATCCACCTAA
cat	F: CTCCTGATGTGGCCCGATAC R: TCAGATGCCCGGCCATATTC
Cu/Zn SOD	F: CAACACAAACGGCTGCATCA R: TTTGCAACACCACTGGCATC
Mn sod	F: AGCGTGACTTTGGCTCATTT R: ATGAGACCTGTGGTCCCTTG
nrf2	F: AACGAGTTCTCCCTTCAGCA R: ATTTTGTCGCCGATTTTGTC
nqo1	F: CACAGGATTGCCTTCAGC R: CGTGTATGCAGGAGACCAGA
tp53	F: CAGGCCCATCCTCACAAT R: ATTCTTCGTCCTTCACCA
gapdh	F: TGAGGTTAAGGCAGAAGGCG R: CCCTTAATGTGAGCAGAAG
cmlc2	F: ATACAGGAGTTTAAGGAG R: GCAACTGAGTATGAAGTTTATTA
nppa	F: ACAGAGACCGAGAGGAAG R: AGGGTGCTGGAAGACCCTAT

Table S2. Significantly enriched GO terms after exposure to PEDOT: PSS

GO ID	Term Type	Description	Gene_names	Up	Down
GO:0032922	biological process	circadian regulation of gene expression	per1b;nr1d2a;nr1d1;per1a;nocta;egr1;ciartb	0	7
GO:0015272	molecular function	ATP-activated inward rectifier potassium channel activity	kcnj1a.6;kcnj1a.4;kcnj1a.3;kcnj1a.5	0	4
GO:0007623	biological process	circadian rhythm	per1b;nr1d2a;nr1d1;per1a;nocta;egr1;ciartb	0	7
GO:0033559	biological process	unsaturated fatty acid metabolic process	si:dkey-1k2.7;elovl1a;elovl7b;ptgs2b;ptgs2a	0	5

Table S3. Significantly enriched KEGG pathways after exposure to PEDOT: PSS

KEGG Pathway ID	Description	Gene_names	Up	Down
map04657	IL-17 signaling pathway	cebpb;fosb;mmp13a;ptgs2a;ptgs2b; cxcl19;il1b;fosaa;fosl1a;fosab	0	10
map04380	Osteoclast differentiation	fosb;il1b;fosl2;stat1b;junbb;acp5a;cyldb; junba;fosaa;fosl1a;fosab	0	11
map04668	TNF signaling pathway	cebpb;il1b;ptgs2a;junbb;ptgs2b;creb3l3a; junba;fosaa;fosab	1	8
map04960	Aldosterone-regulated sodium reabsorption	kcnj1a.6;sgk1;kcnj1a.4;kcnj1a.3;kcnj1a.5	0	5
map04625	C-type lectin receptor signaling pathway	egr2a;il1b;stat1b;ptgs2a;ptgs2b;caspb; cyldb;egr3	0	8
map04917	Prolactin signaling pathway	gck;stat1b;cishb;cish;fosaa;fosab	1	5
map04928	Parathyroid hormone synthesis, secretion and action	mmp13a;egr1;prkacba;creb3l3a;pth3r; fosaa;fosab	3	4

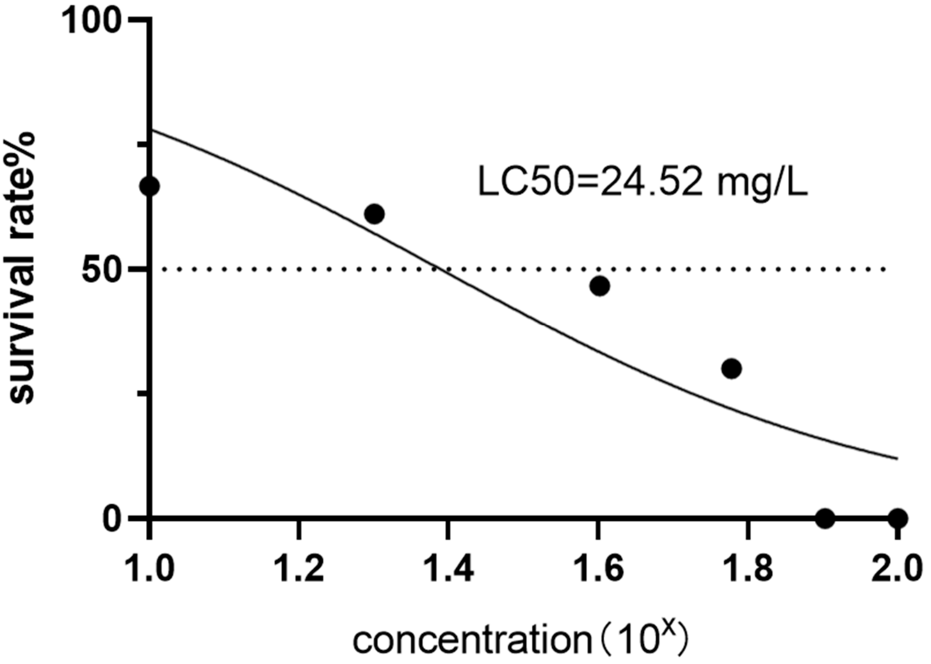

Figure S1. The curve showing the LC_{50} value of zebrafish larvae after exposure to PEDOT: PSS for 96 h.
